# From Understanding to Appreciating Music Cross-Culturally

**DOI:** 10.1371/journal.pone.0072500

**Published:** 2013-09-04

**Authors:** Thomas Hans Fritz, Paul Schmude, Sebastian Jentschke, Angela D. Friederici, Stefan Koelsch

**Affiliations:** 1 Department of Neurology, Max Planck Institute for Human Cognitive and Brain Science, Leipzig, Germany; 2 Department of Educational Sciences and Psychology, Freie Universität, Berlin, Germany; 3 Institute for Psychoacoustics and Electronic Music, University of Gent, Gent, Belgium; 4 Department of Nuclear Medicine, University of Leipzig, Leipzig, Germany; 5 Department of Neuropsychology, Max Planck Institute for Human Cognitive and Brain Science, Leipzig, Germany; University of Tokyo, Japan

## Abstract

It has long been debated which aspects of music perception are universal and which are developed only after exposure to a specific musical culture. Here we investigated whether “iconic” meaning in Western music, emerging from musical information resembling qualities of objects, or qualities of abstract concepts, can be recognized cross-culturally. To this end we acquired a profile of semantic associations (such as, for example, fight, river, etc.) to Western musical pieces from each participant, and then compared these profiles across cultural groups. Results show that the association profiles between Mafa, an ethnic group from northern Cameroon, and Western listeners are different, but that the Mafa have a consistent association profile, indicating that their associations are strongly informed by their enculturation. Results also show that listeners for whom Western music is novel, but whose association profile was more similar to the mean Western music association profile also had a greater appreciation of the Western music. The data thus show that, to some degree, iconic meaning transcends cultural boundaries, with a high inter-individual variance, probably because meaning in music is prone to be overwritten by individual and cultural experience.

## Introduction

To our knowledge, there is no musical tradition in which music has propositional semantics, unless it imitates language (e.g., drumming languages, [1], [2], [3]). In Western culture, however, different musical pieces do vary with respect to the associations they evoke. Evidence from semantic priming studies indicates that music can prime representations of meaningful concepts: It was shown that the N400 event related potential, which is considered to be an electrophysiological index of semantic information processing, was modulated by musical information preceding a target word [4], [5], [6], [7], [8], [9].

Musical meaning can be divided into at least two fundamentally different classes, extra-musical (1) and intra-musical (2).

1. Extra-musical meaning is created with reference to an object, concept or state of the extra-musical world. Note that there have been several approaches of how quality of sound can be mapped to meaning [10], [11], [12], [13], [14]. Below we adapt the one of Karbusicky [15] and Koelsch [16], who further differentiate the extra-musical meaning domain into three categories, in correspondence to the semiotics introduced by Charles Sanders Peirce [17]. Peirce proposed a categorization of semantic meaning in *icons*, *indices*, and *symbols* where every sign refers either through similarity to its object (icon – e.g. a portrait, diagram, metaphor that resemble an object), through factual connection (index – e.g. a depiction of smoke coming out of a building as an index for fire), or through interpretive habit or norm of reference (symbol – most words are symbols).
  - (1 a) Meaning emerging from common patterns or forms, such as musical sound patterns that resemble sounds or qualities of objects (e.g. regularly ascending melodies are often easily associated with staircase), concepts, and states.This sign quality is reminiscent of Charles Sanders Peirce's “iconic” sign quality [17]; in language, this sign-quality is also referred to as *onomatopoeic*.
  - (1 b) Meaning can also emerge from “indexical sign quality”, signalling inner states (e.g., happy or sad) [16]. Evidence showing that different emotional expressions intended or commonly felt in Western music (happiness, sadness, fearfulness) can be recognized by individuals who had never before listened to Western music suggests that at least some musical indexical sign qualities (some emotional expressions in Western music) are understood universally [18].
  - (1 c) “Symbolic sign quality”: There is meaning due to explicit (or conventional) extra-musical associations (e.g., any national anthem).
2. Intra-musical meaning is created due to reference of one musical element, or group of musical elements, to another musical element, or group of musical elements [19].

The differentiation of iconic and indexical extra-musical sign quality has received neuropsychological support by findings indicating that patients with fronto-temporal lobe dementia (FTLD) have a selective impairment for the recognition of indexical musical sign quality (showing difficulties to recognize musical expression of happiness or sadness), while the recognition of iconic musical sign quality is intact (e.g., recognition of a musical excerpt sounding like raindrops) [20]. On the other hand, a patient with Alzheimer's disease showed preserved recognition of indexical musical sign quality while the recognition of famous tunes was impaired [21].

The investigation of musical universals with Western music stimuli would ideally require participants who are completely naïve to Western music. Even individuals from non-Western cultures who have only listened to Western music occasionally, and perhaps without paying explicit attention to it (e.g., while listening to the radio or watching a movie) do not qualify as participants because musical knowledge is typically acquired implicitly, and is thus even shaped through inattentive listening experience [22]. The individuals investigated in the present study belong to the Mafa, one of approximately 250 ethnic groups that make up the population of Cameroon. They are located in the Extreme North in the Mandara mountain range, where the more remote Mafa settlements do not have electrical supply, and are still inhabited by many individuals who pursue a traditional lifestyle, some of whom have not been exposed to Western music.

Interestingly, the Mafa do not have a word for music because all musical activity is an integral part of actions or rituals [18]. This indicates that for the Mafa, music is highly ritualistic, interpersonal, and symbolic and Mafa music is rather unlikely to be appreciated for its iconic sign-quality. Nonetheless, previous research with the Mafa has indicated that listeners who were naive to Western music could recognize emotional expressions in Western music (“indexical” meaning) [18].

This research also indicated that an increased capability of the Mafa to recognize emotional expressions in Western music correlated with their appreciation of Western music and their aversion to reversed versions of this music. A temporal distortion of the music by reversion (playing it backwards) obviously entails a strong distortion of musical structure and accordingly of musical meaning encoded in the music. It is thus safe to assume that those listeners who perceived indexical extra-musical meaning in the Western music (emotional expressions) were especially sensitive to a distortion of such musical meaning. The expression of emotions is a quite specific case of musical meaning, however, also because emotional expression in music can be emotionally contagious in the listener.

To our knowledge, iconic meaning in music has neither been examined for its perceptual universality, nor has its link to music appreciation yet been conclusively investigated. Here we addressed whether Western music can convey meaning universally due to iconic sign quality of extra-musical meaning in non-emotional expressions. We also investigated whether a more Western pattern of semantic associations with iconic musical signs would increase the appreciation for Western music and the aversion to a temporal disruption of Western music by playing it backwards (similar to a more Western-like pattern of indexical music association [18]).

## Methods

### Participants

**Figure 1:**
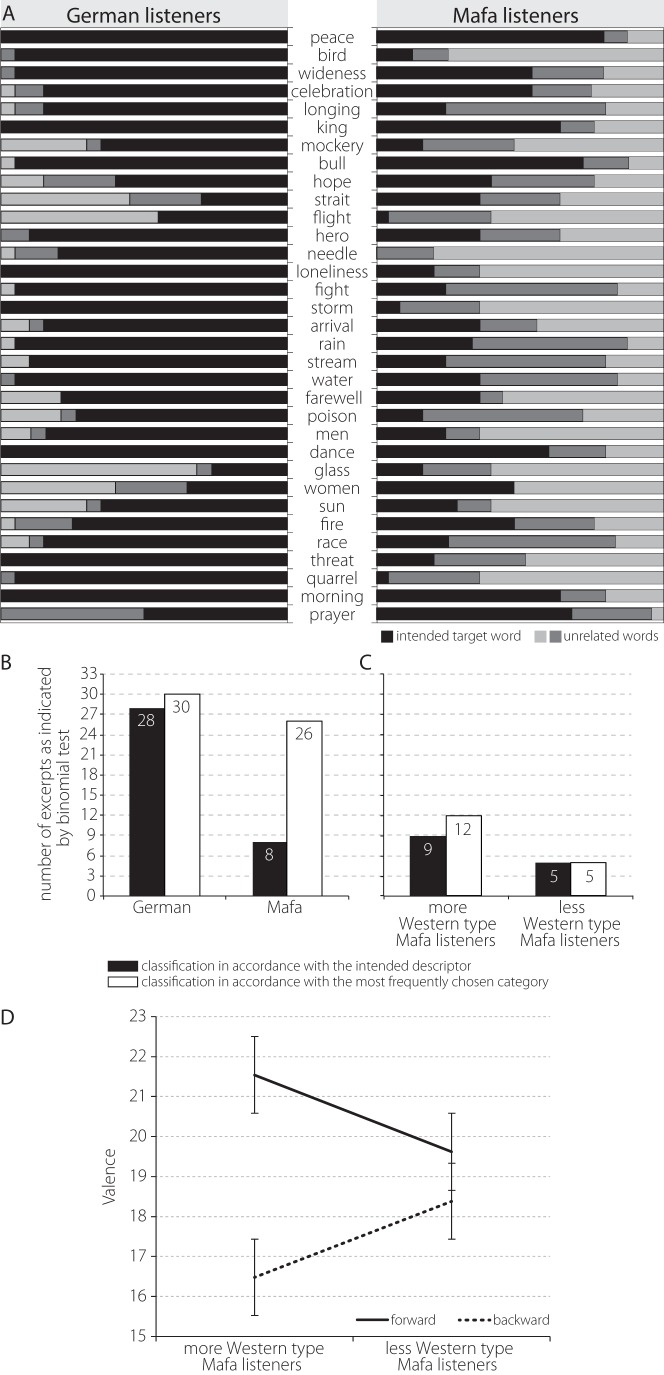
Association profiles. Figure 1A shows the mean association profiles of German and Mafa listeners for the Western musical pieces presented. The middle row indicates what the musical pieces were intended to express, the bars in each line correspond to the three words in each trial that the participants could choose (black – intended descriptor, light and dark grey – unrelated words). Figures 1B and 1C show the absolute number of words (out of a total of 33) classified in accordance with the intended descriptor (in black), as indicated by a binomial test. It furthermore shows the number of words that were chosen in accordance with the most frequently chosen category(as indicated by a binomial test) and thus the extent to which there was agreement within the group in the choice of words. Figure 1B shows these data for all Germans and Mafa, Figure 1C for the more Western type listeners and the less Western type listeners that performed both experiments (N = 12). Figure 1D shows the interaction effect observed in the ANOVA, depicting the mean valence ratings for forward and backward music excerpts, error bars showing standard errors.

Twenty-five Mafas (12 males, ∼37 to ∼80 years old, *M* = 58 years) unfamiliar with Western music, and twenty Westerners (non-musicians, 10 males, 40 to 68 years old, *M* = 52.1 years) participated in Experiment 1 (investigation of iconic meaning in music). Criteria for the selection of Western listeners were that they were not familiar with African music and matched the age range of the Mafa participants approximately. In a standard procedure all Mafa participants gave verbal consent to participate in the study, no written consent was obtained due to illiteracy of the Mafa subjects. The complete explanation of the procedure and the consent of the participants was witnessed and certified by a local assistant. The study procedure (including oral consent) was approved by the ethics committee of the University of Douala, and an official research permit of the government of Cameroon was obtained. German listeners gave written consent. The study procedure was approved by the ethics committee of the University of Leipzig, The study was conducted according to the Declaration of Helsinki. In Experiment 2, 12 Mafa who also participated in Experiment 1 rated how pleasant/unpleasant they perceived Western music and its reversed versions (Figure 1C, D; 10 males; ∼37 to ∼70 years, *M* = 57.1 years).

### Stimuli and Experimental Design

#### Experiment 1

Stimuli were short pieces of Western instrumental music that had previously been used/developed to study the N400 event related response, where they were extensively tested and presented as prime stimuli in combination with target and non-target words [4]. 33 stimuli were presented using the Presentation software (version 0.70, www.neuro-bs.com) on a laptop (charged by a mobile solar electricity facility). After each presentation of a musical excerpt, three nouns were presented of which the participants had to choose the one that they thought best fit the musical piece, and indicate this on a three-button interface (for a list see File S1). All instructions and stimuli were presented over headphones. Only nouns were presented that had corresponding concepts in the Mafa language (words such as “staircase”, for example, were excluded from the study because the Mafa who live traditionally in the Mandara mountain range do not use stairs). The Mafa listeners, who were unaccustomed to handling the button interface were supported by specially trained assistants who, during the presentation of the words, would point to the respective buttons, and immediately after the presentation of the words would again quickly indicate which word was associated with which button. The assistants were unaware that from a Western point of view one of the words was a descriptor (matching the intended meaning of the excerpt from a Western perspective). They were required to stay passive and only repeat the words while pointing to each button, and they were all unaccustomed with the type of Western music presented. This procedure worked smoothly for the Mafa, whereas the Western listeners did not require such assistance. Note that during the experiment, the stimuli were not audible to the experimenter to avoid response biases.

#### Experiment 2

The stimulus material included 14 Western music pieces that were joyful instrumental dance music from the past four centuries (acoustic examples in additional online material) that were successfully utilized as stimulus material in previous studies [18], [23], [24], [25]. The excerpts were chosen to include a variety of different musical styles (including e.g. various classic periods, tango, jazz, folk music of different traditions). The stimulus material also comprised reversed counterparts of the original excerpts (where the sound file was played backwards). Each stimulus was presented in three versions, 2, 10, and 30 seconds long, so that each condition comprised 42 items. Stimuli and instructions were presented using Presentation software. The participants were asked to indicate their appreciation/dislike of the music on a continuous scale with a slider interface.

### Statistical evaluation

For Experiment 1, statistical analyses using binomial tests, explored the response pattern to each of the musical excerpts and within each participant group (Mafa and Westerners): For each song and separately for each group, we determined the probability of observing the number of choices for either the intended descriptor category (according to [4]) or the accordance with the most frequently chosen category. That is, given the three possible choices (i.e., p = 1/3 for each choice) we determined the probability for obtaining the observed number of choices within each group. If the probability exceeded 0.95 (i.e. having an error probability α<0.05) this was counted as response in accordance with the intended or the most commonly chosen category. The first analysis determined whether the frequency with which the intended descriptor category was chosen exceeded chance level (α<0.05; Figure 1B, black bars). Another similar binomial test evaluated whether the most commonly chosen category for each excerpt was selected more often than expected by chance (α<0.05; Figure 1B, white bars), indicating the number of words chosen in accordance with the most frequently chosen category. The rationale for the second analysis was that Mafa listeners might not have chosen the descriptor category although they agreed on one choice among the three possible answers.

To investigate if a capability to decode iconic meaning from Western music changes musical appreciation for Western music we performed an analysis where we relate the findings of both experiments. For this, we divided the Mafa participants into two groups, dependent on how many music excerpts each participant classified according to the intended (“Western”) descriptor. They were divided, so that one of the groups was above the median and the other below the median. Note that only 12 participants performed both experiments so that each Mafa subgroup contained only 6 participants, which implies a rather low statistical power. A group of binomial tests similar to those described above and depicted in Figure 1B were conducted that explored the response pattern to each of the musical excerpts and within each participant group (more Western type listeners, less Western type listeners; depicted in Figure 1C). An ANOVA with the factors „group“ (more/less Western listeners) and „direction” (forward/backward) was calculated (Figure 1D).

## Results

### Experiment 1

We used a classification paradigm where in each trial a musical piece was presented, and then participants had to choose the best fit (descriptor) out of three words that were presented at the end of the music presentation (Figure 1A). The Western and Mafa association profiles did not significantly overlap, but were consistent for each group. We calculated this, comparing the association patterns of the Western group and the Mafa listeners with a chi-square goodness of fit test, where the Western association pattern (as derived from previous experiments; Koelsch, Kasper et al., 2004) was used as the relative frequency, and the association pattern of the Mafa listeners was used as the absolute frequency. The chi-square goodness of fit test showed that the profiles were significantly different from each other (*p* = 0.015).

### Experiment 2

We presented instrumental Western music excerpts and the same excerpts played backwards to both Mafa and Western individuals (see File S1 for sound examples). This paradigm was already reported in a previous study [18], and has the advantage that it allowed us to compare naturalistic music excerpts (i.e., ecologically valid stimuli) to their manipulated (reversed) counterparts. Results indicate that the Mafa listeners rated the original Western music excerpts as more pleasant than the reversed ones (*p* = 0.038; effect size d = 0.86), using a two-tailed two sample t-test with 12 males and 42 stimuli.

### Is the capacity to decode iconic meaning from Western music related to the appreciation of Western music?

To investigate this question, we will relate the results from Experiments 1 with those from Experiment 2. The valence-differences between the original and the backward music excerpts were compared with the degree to which Mafa and Western listeners showed a typical Western pattern of music semantic association. Mafa participants were divided into two groups depending on the number of music excerpts they categorized according to the intended (“Western”) descriptors. An ANOVA with the factors group and direction of music (forward, backward) revealed a main effect of “direction” (F = 24,994; p<0,01): the original forward played songs got higher pleasantness ratings than the excerpts played backward. The analysis did not show a main effect of the factor “group”. An interaction effect group*direction was observed (F = 9,234; p<0,01; see Fig. 1D): Forward played songs received higher and backward played song lower valence ratings in the group of listeners that chose descriptions more in accordance with intended (“Western”) categories, whereas there was hardly a difference in the valence ratings in the other group of listeners that chose descriptions less in accordance with intended (“Western”) categories.

## Discussion

Western and Mafa listeners show culture-specific profiles of how the musical excerpts are associated with word meaning (Figure 1A). Figure 1B shows that Germans more strongly associate the musical pieces with the intended strongest association as previously evaluated for German listeners [4], while Mafas on average do not perform above chance level (1/3) in this respect. This indicates that the cultural background has a major influence on the verbal associations evoked by the music.

A frequency analysis showing that certain words are likely to be associated with musical sounds because they are related to Mafa rituals (for example bull) suggests that the Mafa listeners observed a conceptual similarity between the musical sounds they perform during their rituals (which are usually accompanied by musical performances), and the unknown Western musical excerpts presented to them during the experiment (over headphones). Because music occurs in ritual contexts in Mafa life, in a choice paradigm the Mafa would thus probably settle disproportionately on the closest generic analogs among them, when presented with stimuli they recognize as musical. This would probably include celebration and dance, which is also the case in other culture concepts of music. E.g. the origin of our term music in its original Greek (mousiké) included melody, dance and poetry, the term ngoma of the Bantu language group subsumes drumming, singing, dancing and festivity, and the Blackfoot word saapup denotes singing, dancing, and ceremony in a single concept [26], [27], [28]. This is in accord with previous theoretical considerations about music perception in autochthonous peoples, arguing that they probably generalize musical sound although they often do not have a word for music [29].

Interestingly, Figure 1B also shows that the Mafa display a similar association profile within their own cultural group, associating 26 out of the 33 musical pieces according to the most frequent association of their cultural group, i.e. the nouns that were picked by a significant majority of the Mafa in each trial (white bars). This means that although the pattern of meaning association in Mafa listeners is different from that of Western listeners, it displayed a pattern of association that was consistent within the Mafa group. This demonstrates that the types of associations evoked by Western music are systematically shaped by enculturation and, unlike associations evoked by basic emotional expressions in music, vary strongly between cultures.

Emotional expression in music is a special case of musical semantics: that emotional expression can be contagious, seems to be cross-culturally perceived in other modalities [30], and has also been shown to transcend modalities [31]. A cross-cultural capacity to identify emotional expressions in Western music may be partly due to a more general capability to recognize nonverbal patterns of emotional expressiveness [32] such as emotional prosody [33]. Note that Western music has been observed to mimic emotional prosody as a means of emotional expression [34]. Furthermore, similar emotion-specific acoustic cues are used to communicate emotion in both speech and music [35], [36].

Western music is the result of a long cultural integration process, which has probably promoted the cultural transmission of musical features such as emotional expression that may well be common perceptual denominators between many music cultures [37]. The current data show that this is not necessarily the case for all aspects of iconic meaning in music. The different association profiles of the Mafa (compared to Westerners) are possibly partly due to an overriding of iconic meaning by the symbolical associations the Mafa have with many of the unknown music excerpts due to cultural associations of their own (e.g. such that musical sound is closely associated with their own music-accompanied rituals). On the other hand, the different association profiles of the Mafa (compared to Westerners) are possibly also due to the fact that the meaning of the nouns used in Experiment 1 (and probably most existing nouns) is not identical across different cultures. It is quite interesting to note that in the case of some of the Mafa associations, such as for example their choice of “peace”, the Mafa agree pretty well with the intended descriptors, raising the possibility that those musical excerpts in fact manage to convey their intended meaning even across the enculturation barrier.

The appreciation of Western music across cultures was investigated in Experiment 2, which explored how pleasant/unpleasant it was for participants to listen to Western music excerpts and their reversed (played backwards) counterparts. Previous evidence showed that Mafa who were better at identifying emotional expressions in Western music (happiness, sadness, fearfulness), were also more sensitive to a temporal distortion of Western music [18].This gave rise to the idea that, more generally, all those listeners (unfamiliar with Western music) who are better at identifying meaning in Western music are also more sensitive (in terms of appreciation) to its temporal corruption by playing it backwards. Here we tested whether this holds for iconic musical meaning, resembling properties of objects and concepts rather than emotional psychological states. An ANOVA was computed to test for whether a capacity to decode iconic meaning from Western music entails a difference in the appreciation of Western music (relating the results from Experiments 1 and 2). This ANOVA revealed that those Mafa listeners whose iconic meaning association profile for the Western music was more similar to the average association profile of the Western listeners (as indicated by the number of music excerpts they categorized according to the intended (“Western”) descriptors) also showed a larger difference in their valence ratings for the forward played compared to the backward played musical pieces (see also Figure 1D). This indicates that the capability to decode iconic meaning in music is related to appreciation differences in the listeners, similar to how the capability to decode indexical meaning (more specifically emotional expressions) is related to appreciation differences in the listeners [18] (note however, that only 12 participants were included in the analysis relating Experiments 1 and 2, which implies a low statistical power). This suggests that a possible contagious effect of musical emotional expression in the previous study was not mediating the previously observed effects (but rather the degree of meaningfulness of the music).

A limitation to the present paradigm is that reversed playing changed the nature of the stimuli in ways that go beyond structural changes in the sequence of the music. Beyond musical structure, it also affects the characteristics of instrument decay and a fading of ambient reverberations to which listeners are accustomed so that the reversed versions may sound more unnatural. This may be relevant because it is conceivable that some individuals may have been generally less attentive, motivated, or aware of their experimental task, and that this has influenced both their valence ratings and their performance in the association profiles. Another limitation is that we pursued a psychological rather than anthropological approach in our investigation. There is a characteristic difference in perspective between these two disciplines on the issue if transcultural study of cognition is primarily a study of codes (sets of rules for the appropriate construction and interpretation of a message), or of intellective processes (which can account for the behavioral performance of individuals). It has been argued that the psychologist in transcultural studies will typically “force” subjects to do unusual tasks, for which a semantic differential approach would probably qualify [38]. From this point of view, any kind of shared behavior would be the result of an interesting intellectual process. From an anthropologists' perspective, asking inappropriate questions would rather be an inefficient strategy, because in this field in order to investigate the nature of a coding system, informants must be kept within this system [38].

Importantly, the current finding also shows that iconic meaning in music can transcend cultures, but that there is a high variability in the individual inclination to decode such iconic musical meaning. On what did the Mafa individuals base their propensity to decode iconic meaning from previously unheard music? There is evidence that musical changes in amplitude, pitch contour, pitch intervals, attack rate, articulation and tempo are closely associated with human movements in space [39], [40], [41]. Because a multitude of nouns evoke either motor or Gestalt relations (for example how one would use a tool, or how one would expect a king to move (dignified)), such movement associations would successfully help to narrow down a choice for a corresponding musical piece. Note that a correspondence between sound and motor/Gestalt relations is established and trained early in life during mother-infant interaction [42], [43], which is considered to be universally similar with respect to rhythm and intonation [42], and is at work probably even earlier, before birth [44], [45].

## Supporting Information

File S1 **Tables listing all the word triplets presented in German, Mafa (using the International Phonetic alphabet IPA), and an English translation.**
(PDF)
