# Supplementary material for: From Understanding to Appreciating Music Cross-Culturally
Source: PLoS One. 2013 Sep 4;8(9):e72500. doi: 10.1371/journal.pone.0072500 (PMC3762814; doi:10.1371/journal.pone.0072500)
Supplement: File S1 — Tables listing all the word triplets presented in German, Mafa (using the International Phonetic alphabet IPA), and an English translation. (PDF) [file pone.0072500.s001.pdf]

Supplementary tables listing all the word triplets presented in German, Mafa (using the International Phonetic alphabet IPA), and an English translation.

Table S1.

English

sound	word 1	word 2	word 3
peace	storm	quarrel	peace
Bird	bird	bull	longing
wideness	strait	mockery	wideness
celebration	fight	celebration	stream
longing	longing	race	king
King	king	needle	bird
mockery	rain	mockery	joy
Bull	bird	bull	poison
hope	impatience	hope	fire
strait	wideness	hero	strait
flight	dance	sun	flight
hero	poison	loneliness	hero
needle	king	arrival	needle
loneliness	fire	loneliness	celebration
fight	fight	celebration	grief
storm	peace	storm	needle
arrival	farewell	impatience	arrival
Rain	rain	mockery	men
stream	stream	threat	race
water	water	sun	dance
farewell	arrival	flight	farewell
poison	hero	poison	women
men	women	men	glass
dance	dance	flight	morning
glass	morning	glass	storm
women	men	strait	women
Sun	water	sun	threat
Fire	loneliness	farewell	fire
race	race	longing	water
threat	stream	threat	hope
quarrel	prayer	peace	quarrel
morning	morning	glass	fight
prayer	quarrel	wideness	prayer

Table S2.

Mafa

sound	word 1	word 2	word 3
[pam'bai]	[n'tɒmbɔn'dɒ]	[ɤsa'ɒn]	[pam'bai]
['biak]	['biak]	[ma'rai]	['ski:ma'nangakiɛ]
[nhħu'aa]	['yxeɛ]	[ngwe'ʃe am'da]	[nhħu'aa]
['œgwala'la]	[ɛ̃̏gijobi:]	['œgwala'la]	[vweɪ'jɳ]
['ski:ma'nangakiɛ]	['ski:ma'nangakiɛ]	[œ'xi]	['bi:]
['bi:]	['bi:]	[lɛ'pɛr]	['biak]
[ngwe'ʃe am'da]	[jʌmiʒ'glɛ]	[ngwe'ʃe am'da]	['ngfe]
[ma'rai]	['biak]	[ma'rai]	[n'dɛre]
[i'ʒɛrskwai]	ungeduld	[i'ʒɛrskwai]	[vɑ̃'bwap]
['yxeɛ]	[nhħu'aa]	[n'do saje'daʎ]	['yxeɛ]
[n'mandɛdʒɪmɛ'e]	[ɤge'tʃe]	['bats]	[n'mandɛdʒɪmɛ'e]
[n'do saje'daʎ]	[n'dɛre]	[noma'na nsa'a agn'ɐ]	[n'do saje'daʎ]
[lɛ'pɛr]	['bi:]	['ɤndike]	[lɛ'pɛr]
[noma'na nsa'a agn'ɐ]	[vɑ̃'bwap]	[noma'na nsa'a agn'ɐ]	['œgwala'la]
[ɛ̃̏gijobi:]	[ɛ̃̏gijobi:]	['œgwala'la]	[nlaɪ'e]
[n'tɒmbɔn'dɒ]	[pam'bai]	[n'tɒmbɔn'dɒ]	[lɛ'pɛr]
['ɤndike]	[n'de]	[ha'ʃɛʀɛɛtipaˈi]	['ɤndike]
[jʌmiʒ'glɛ]	[jʌmiʒ'glɛ]	[ngwe'ʃe am'da]	['ɛ̃gura]
[vweɪ'jɳ]	[vweɪ'jɳ]	['skɥiʀ ʊʊ 'i:tba]	[œ'xi]
[ˈjʌm]	[ˈjʌm]	['bats]	[ɤge'tʃe]
[n'de]	['ɤndike]	[n'mandɛdʒɪmɛ'e]	[n'de]
[n'dɛre]	[n'do saje'daʎ]	[n'dɛre]	[n'gɒz]
['ɛ̃gura]	[n'gɒz]	['ɛ̃gura]	[gwala'ba]
[ɤge'tʃe]	[ɤge'tʃe]	[n'mandɛdʒɪmɛ'e]	['prɛk]
[gwala'ba]	['prɛk]	[gwala'ba]	[n'tɒmbɔn'dɒ]
[n'gɒz]	['ɛ̃gura]	['yxeɛ]	[n'gɒz]
['bats]	[ˈjʌm]	['bats]	['skɥiʀ ʊʊ 'i:tba]
[vɑ̃'bwap]	[noma'na nsa'a agn'ɐ]	[n'de]	[vɑ̃'bwap]
[œ'xi]	[œ'xi]	['ski:ma'nangakiɛ]	[ˈjʌm]
['skɥiʀ ʊʊ 'i:tba]	[vweɪ'jɳ]	['skɥiʀ ʊʊ 'i:tba]	[i'ʒɛrskwai]
[ɤsa'ɒn]	[ɱtser'i:]	[pam'bai]	[ɤsa'ɒn]
['prɛk]	['prɛk]	[gwala'ba]	[ɛ̃̏gijobi:]
[ɱtser'i:]	[ɤsa'ɒn]	[nhħu'aa]	[ɱtser'i:]

Table S3.

German

Sound	word 1	word 2	word 3
Frieden	Sturm	Streit	Frieden
Vogel	Vogel	Stier	Sehnsucht
Weite	Enge	Spott	Weite
Fest	Kampf	Fest	Bach
Sehnsucht	Sehnsucht	Rennen	Koenig
Koenig	Koenig	Nadel	Vogel
Spott	Regen	Spott	Freude
Stier	Vogel	Stier	Gift
Hoffnung	Ungeduld	Hoffnung	Feuer
Enge	Weite	Held	Enge
Flucht	Tanz	Sonne	Flucht
Held	Gift	Einsamkeit	Held
Nadel	Koenig	Ankunft	Nadel
Einsamkeit	Feuer	Einsamkeit	Fest
Kampf	Kampf	Fest	Trauer
Sturm	Frieden	Sturm	Nadel
Ankunft	Abschied	Ungeduld	Ankunft
Regen	Regen	Spott	Maenner
Bach	Bach	Bedrohung	Rennen
Wasser	Wasser	Sonne	Tanz
Abschied	Ankunft	Flucht	Abschied
Gift	Held	Gift	Frauen
Maenner	Frauen	Maenner	Glas
Tanz	Tanz	Flucht	Morgen
Glas	Morgen	Glas	Sturm
Frauen	Maenner	Enge	Frauen
Sonne	Wasser	Sonne	Bedrohung
Feuer	Einsamkeit	Abschied	Feuer
Rennen	Rennen	Sehnsucht	Wasser
Bedrohung	Bach	Bedrohung	Hoffnung
Streit	Gebet	Frieden	Streit
Morgen	Morgen	Glas	Kampf
Gebet	Streit	Weite	Gebet
